# Photoinduced acute exanthematous pustulosis caused by dicloxacillin and exposure to sunlight

**DOI:** 10.1002/ccr3.2681

**Published:** 2020-02-14

**Authors:** Rikke M. Nielsen, Kristine A. Pallesen

**Affiliations:** ^1^ Department of Dermatology and Allergy Centre Odense University Hospital Odense Denmark

**Keywords:** Acute generalized exanthematous pustulosis, photoinduced acute exanthematous pustulosis

## Abstract

Photoinduced acute exanthematous pustulosis is a rare condition; only few cases of photo‐AEP have been described previously with drugs such as norfloxacin, ciprofloxacin, and enoxacin. In this case, the reaction is seen after intake of dicloxacillin.

## Graphical abstract

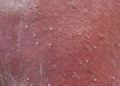

## 1 CASE REPORT

A seventy‐five‐year‐old man was admitted to hospital with a 2‐day history of rash. Eight days before admission, he was prescribed oral dicloxacillin 1000 mg three times a day as treatment for folliculitis in the scalp. In the same period, he had been bicycling more than 100 km during a week. After 1 week, a sudden eruption of generalized erythematous rash with white cutaneous pustules developed in the face spreading to the rest of the body. He also began to feel fever chills. Dicloxacillin was discontinued, and treatment with prednisolone 12.5 mg once daily and fexofenadine 180 mg twice daily was initiated. He was known with a past medical history of hypertension and allergic rhinitis and was bypass operated twice, but he had no previous history of psoriasis or other skin conditions. He had a warning registration in the medical journal for a contrast agent due to generalized pruritus.

**Figure 1:**
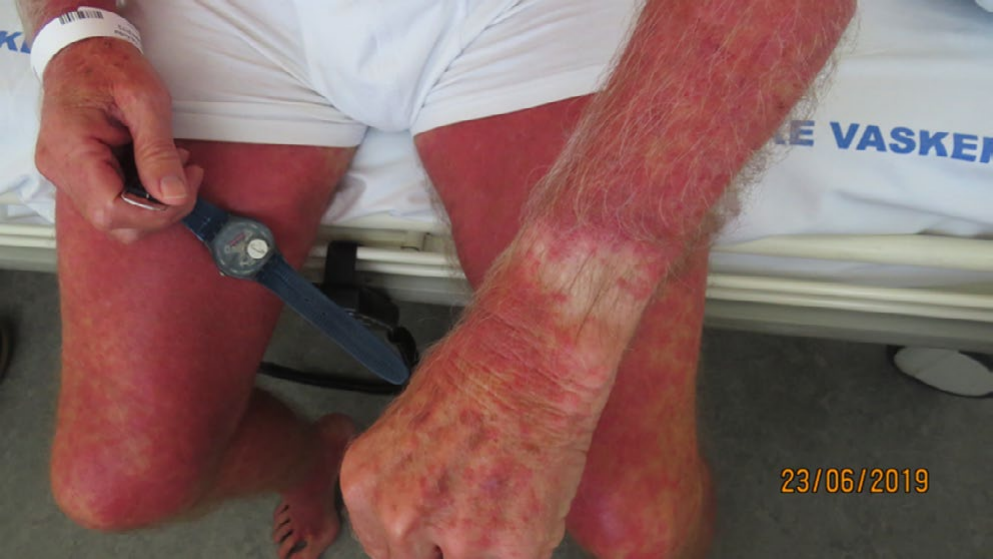
Acute exanthematous pustulosis on sun‐exposed skin. Skin under watch was uninvolved

**Figure 2:**
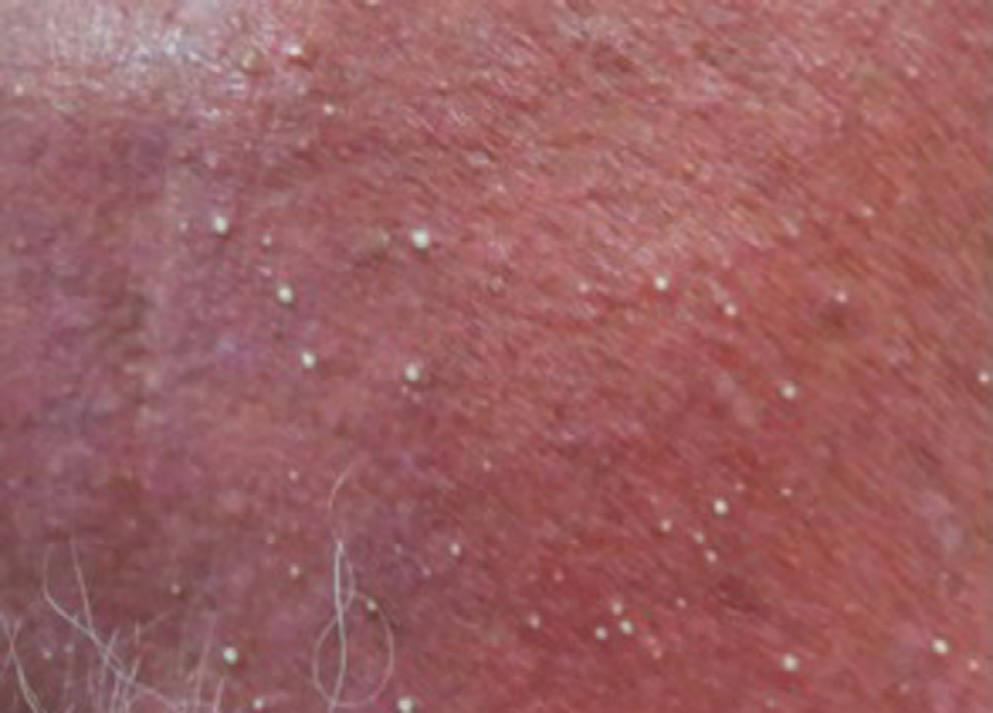
Generalized erythematous skin with white pustules

Physical examination revealed tachycardia (heart rate 160/min), blood pressure 156/79 but normal respiratory frequency and saturation. By admission, no fever was present. Examination of the skin revealed a generalized erythematous skin eruption where the skin had been exposed to sunlight, leaving the skin under his watch and clothes (shorts area) completely uninvolved with a sharp boundary to exposed skin (Figure 1). Dozens of white pinhead sized pustules were seen on the chest (Figure 2). The pustules spread within few days to involve shoulders, abdomen, upper back, and legs. There was no involvement of mucous membranes.

Laboratory studies revealed leukocytosis (leukocytosis of 25.1 × 10^9^/L, neutrophils 22.8 × 10^9^/L) and C‐reactive protein (CRP) of 145 mg/L but with normal liver and kidney function. During admission, fever developed (38.9 Celcius) and the infection counts increased (CRP 193, leukocytes 26.8). A skin biopsy from the chest was not fully representative of the clinical diagnosis. It was described as acute folliculitis showing spongiosis with neutrophil exocytosis and papillary edema, and inflammation with lymphocytes, macrophages, and neutrophils. The patient was treated with topical corticosteroids and systemic prednisolone 25 mg for a few weeks with good response, and upon checkup two weeks later, he had recovered completely.

## 2 DISCUSSION

Acute generalized exanthematous pustulosis (AGEP) is a severe cutaneous reaction first described in 1980 by Beylot et al^1^ The disease is caused by acute viral infections, mercury, or medications, often anticonvulsants or antimicrobial drugs such as beta‐lactam antibiotics.^2^ Withdrawing of the offending drug is important, and the disease has a self‐limited course.

An unusual form of AGEP is caused by exposure to ultraviolet (UV) light, for example, natural sunlight. Photoinduced acute exanthematous pustulosis (photo‐AEP) is, just like AGEP, characterized by rapid eruption of erythematous skin with sterile, cutaneous pustules, but it is triggered by exposure to UV light in combination with intake of a systemic drug. Beside the cutaneous symptoms, the condition is also followed by fever and leukocytosis.^3^

Few cases of photo‐AEP have been described previously*.* 3, 4, 5 In the previous cases of photo‐AEP, quinolone antimicrobials had been implicated as causative factor.^3^, ^4^, ^5^ Quinolones are well known to have photosensitizing qualities.^6^

We present a rare case of skin eruption due to a combination of prescribed oral dicloxacillin and exposure to sunlight. Dicloxacillin is a narrow‐spectrum beta‐lactam antibiotic. Other diagnoses to consider were bacterial and fungal infection in the skin and pustular psoriasis.

The diagnosis of dicloxacillin‐associated photo‐AEP was made based on the clinical findings and because of the rapid recovery on treatment with prednisolone and without further antibiotic treatment. The biopsy did not support the diagnosis. Halevy et al^7^ made a study including 102 patients with AGEP and evaluated on the histopathological findings, where follicular pustules were found in 23% of the cases. Several reasons can explain why the diagnosis cannot be confirmed in the biopsy, for example, biopsy location, size, or time since debut of eruption.

## CONFLICT OF INTEREST

None.

## AUTHOR CONTRIBUTIONS

Rikke Maria Nielsen: served as primary author of the text and as corresponding author, and is responsible for literature search and submission process. Kristine Appel Pallesen: involved in supervision during manuscript writing, adjusted language, and approved the final version.

## References

[1] Beylot CBP . [Acute generalized exanthematic pustuloses (four cases) (author's transl)]. Ann Dermatol Venereol. 1980;107(1–2):37‐48. 6989310

[2] Guevara‐Gutierrez E , Uribe‐jimenez E , Diaz‐Canchola M , Tlacuilo‐Parra A . Acute generalized exanthematous pustulosis: Report of 12 cases and literature review. Int J Dermatol. 2009;48(3):253‐258. 19261012 10.1111/j.1365-4632.2009.03908.x

[3] Knoell KA , Lynch JM . Photoinduced acute exanthematous pustulosis caused by ciprofloxacin and sunlight exposure. Int J Dermatol. 2009;48(10):1141‐1143. 19775415 10.1111/j.1365-4632.2009.04115.x

[4] Shelley ED , Shelley WB . The subcorneal pustular drug eruption: an example induced by norfloxacin. Cutis. 1988;42:24‐27. 2974410

[5] Gillet‐Terver MN , Modiano P , Barbaud A , Reichert S , Schmutz JL . Photoinduced acute exanthematous pustulosis induced by enoxacin. Nouvelles Dermatologiques. 1998;5:381‐383.

[6] Tokura Y . Quinolone photoallergy: photosensitivity dermatitis induced by systemic administration of photohaptenic drugs. J Dermatol Sci. 1998;18(1):1‐10. 9747656 10.1016/s0923-1811(98)00026-7

[7] Halevy S , Kardaun SH , Davidovici B , Wechsler J . The spectrum of histopathological features in acute generalized exanthematous pustulosis: A study of 102 cases. Br J Dermatol. 2010;163:1245‐1252. 20698849 10.1111/j.1365-2133.2010.09967.x

